# In neonatal resuscitation, the ECG monitor informs. Ventilation saves

**DOI:** 10.1016/j.jped.2026.101532

**Published:** 2026-03-25

**Authors:** Georg M. Schmölzer

**Affiliations:** a Centre for the Studies of Asphyxia and Resuscitation, Royal Alexandra Hospital, Edmonton, Alberta, Canada; b Dept. of Pediatrics, University of Alberta, Edmonton, Alberta, Canada

Accurate and timely heart rate (HR) assessment is central to neonatal resuscitation [1,2]. For decades, clinicians relied on auscultation and pulse oximetry to guide interventions [3,4]. Over the last decade, however, electrocardiographic (ECG) monitoring has been increasingly recommended by the neonatal resuscitation guidelines because it provides faster and more reliable HR detection in the first minutes after birth [1,2,[4], [5], [6]]. The expectation has been intuitive: earlier and more precise HR assessment should translate into better clinical decision-making and improved outcomes. The study by Freire et al. in this issue presents a nine-year cohort from a tertiary Brazilian center provides important real-world insight into whether that assumption holds true [7].

## ECG Adoption: From Recommendation to Routine

The progressive increase in ECG use observed in this cohort mirrors global trends following updates from neonatal resuscitation guidelines and consensus of science and treatment recommendations [1,2]. Evidence from physiologic studies and randomized trials has consistently demonstrated that ECG displays HR more rapidly than pulse oximetry in the delivery room [[8], [9], [10], [11], [12], [13], [14], [15], [16]]. This technological advantage led to guideline recommendations favoring ECG for infants requiring positive pressure ventilation (PPV).

By 2022 in this cohort, ECG use approached universal adoption in infants < 34 weeks and exceeded 90% in those ≥ 34 weeks receiving PPV [7]. This trajectory reflects successful guideline implementation at the systems level.

Yet implementation alone does not guarantee clinical impact.

## Intubation Rates: No Signal of Benefit

The primary clinical question whether ECG use reduces the need for tracheal intubation was not supported by the findings [7]. Intubation rates were similar regardless of ECG use in both gestational age strata. This aligns with emerging international data. Randomized trials in preterm infants have shown that while ECG provides faster HR acquisition, it does not consistently alter rates of intubation or advanced resuscitation interventions room [11,14]. Likewise, implementation studies comparing pre- and post-ECG eras have failed to demonstrate meaningful changes in intubation frequency room [12,13]. Taken together, the accumulating evidence suggests that improved HR detection alone may not be sufficient to modify airway management decisions in the delivery room. Intubation in this context is driven by a complex interplay of ventilation effectiveness, respiratory drive, gestational age, team dynamics, and provider thresholds, not HR data alone.

## The Unexpected Finding: Delayed PPV Initiation

Perhaps the most thought-provoking result of this study is the association between ECG use and delayed initiation of PPV beyond 60 seconds of life in both term and preterm infants. The adjusted analysis showed that ECG use was associated with a significantly higher likelihood of delayed ventilation. Infants monitored with ECG were approximately 2.5–2.7 times more likely to receive PPV after the first minute of life compared with those without ECG monitoring [7].

In practical terms, ventilation began about on average by 10 seconds later in infants monitored with ECG (47 vs 36 seconds), and over one-third (37%) received PPV after the “Golden Minute,” compared with only 14% of infants without ECG monitoring [7]. Regardless of the underlying mechanism, these findings emphasize that establishing effective ventilation within the “Golden Minute” must remain the central priority, and that additional monitoring should support, rather than delay, this critical intervention. This finding deserves careful interpretation within the broader literature. Data on whether ECG affects timing of PPV are inconsistent. Some studies show no delay; others report variability without statistical significance [[11], [12], [13], [14]]. However, this cohort analysis demonstrates a clear association after adjustment for potential confounders.

## Why might this occur?

One plausible explanation lies not in the technology itself but in workflow and human factors. In modern resuscitation environments, attention may shift toward equipment setup and data acquisition. Even when electrode placement is delegated to separate team members, the presence of additional monitoring tasks may subtly influence cognitive load, role clarity, or prioritization of interventions [17]. Another important consideration is documentation fidelity. As centers adopt new technologies, they often simultaneously enhance simulation training, debriefing practices, and quality indicator monitoring. Improved time recording may paradoxically reveal delays that previously went unrecognized rather than newly created.

## The Golden Minute Still Matters

The clinical implications are significant. Evidence consistently demonstrates that initiation of effective PPV within the first 60 seconds, the “Golden Minute”, is critical in apneic newborns [18]. Observational data indicate that every 30-second delay in ventilation is associated with increased mortality risk [19]. Early ventilation stabilizes HR and systemic perfusion, preventing progression to circulatory compromise [20]. If ECG use is associated, directly or indirectly, with delayed ventilation, this raises an important systems-level question: Are we prioritizing monitoring over intervention? Technology should support, not distract from, the primary goal of timely and effective ventilation.

## Reframing the Role of ECG

The growing body of evidence, including this study, suggests that ECG improves the speed and reliability of HR acquisition but does not independently reduce intubation rates or resuscitation intensity [12,13]. Its value may lie more in diagnostic clarity and team communication than in altering immediate intervention thresholds.

ECG provides continuous, objective HR data that may i) reduce uncertainty during ambiguous auscultation findings, ii) enhance team situational awareness, iii) support training and debriefing, and iv) standardize documentation. However, it does not replace the foundational principle of neonatal resuscitation: ventilation is the priority.

## Implementation Science, Not Technology Debate

The question is no longer whether ECG detects HR faster, that is well established. The more pressing question is how to integrate ECG into delivery room workflows without compromising the timeliness of PPV. Future work should focus on i) workflow optimization, ii) human factors and cognitive load analyses, iii) simulation-based implementation trials, and iv) process metrics linked to patient-centered outcomes. Rather than debating the device itself, we must examine how teams use it in practice.

## Conclusion

This large single-center cohort reinforces an important and increasingly consistent message: ECG monitoring in the delivery room enhances heart rate detection but does not appear to reduce tracheal intubation rates and may be associated with delayed initiation of PPV. As neonatal care continues to incorporate advanced monitoring technologies, we must ensure that these tools serve the core objective of resuscitation, rapid establishment of effective ventilation. Precision in measurement is valuable, but precision must never come at the expense of timely intervention.

## Data availability statement

N/A.

## Conflict of interest

The author not have any financial or other interests related to this publication. The general COI include that the author is an Emeritus Task Force Member of the Neonatal Task Force with ILCOR, a Scientific Advisory Member within ILCOR. The author is also on the Neonatal Writing for the American Herat Association. The author also developed the RETAIN Board and Computer neonatal resuscitation game, which are commercially available.
